# Phenotypic Associations Between Linearly Scored Traits and Sport Horse Auction Sales Price in Ireland

**DOI:** 10.3390/ani15152227

**Published:** 2025-07-29

**Authors:** Alison F. Corbally, Finbar J. Mulligan, Torres Sweeney, Alan G. Fahey

**Affiliations:** 1 School of Agriculture and Food Science, University College Dublin, Belfield, D04 V1W8 Dublin, Ireland; 2 School of Veterinary Medicine, University College Dublin, Belfield, D04 V1W8 Dublin, Ireland

**Keywords:** horse price, equine economics, market efficiency, phenotypic traits, price determinant

## Abstract

This study examines the associations between linearly scored phenotypic traits and auction sales prices of young event horses in Ireland, aiming to identify key traits influencing market value. Data from 307 horses sold at public auctions (2022–2023) were analysed using regression analysis, binary optimisation, and Principal Component Analysis (PCA). Regression identified Head–neck Connection, Quality of Legs, Walk length of Stride, and Scope as highly significant predictors of sales price (*p* < 0.001), with Length of Croup, Trot Elasticity, Trot Balance, and Take-off Direction also significant (*p* < 0.05). Optimised regression reduced the number of relevant traits from 37 to 8, streamlining evaluation. PCA highlighted eight principal traits, including Scope, Elasticity, and Canter Impulsion, explaining 61.19% of variance in the first four components. These results demonstrate that specific conformation, movement, and athleticism traits significantly affect auction outcomes. The findings provide actionable insights for breeders and stakeholders, suggesting that targeted selection for high-impact traits could accelerate genetic progress and improve market returns. Furthermore, these traits could underpin the development of economic or buyer indices to enhance valuation accuracy and transparency, with potential application across equestrian disciplines to align breeding objectives with market demands.

## Simple Summary

This study addressed the challenge of understanding which physical and movement features most influence the sales price of young event horses at public auctions in Ireland. It aims to help breeders and buyers make better decisions. By analysing data from 307 horses sold between 2022 and 2023, the researchers identified that only a small number of traits—such as the connection between the head and neck, the quality of the legs, the length of the horse’s stride when walking, balance and elasticity in movement, the length of the croup (the horse’s hindquarters), and especially the horse’s “scope” (its ability to jump well)—were strongly linked to higher prices. The study reduced the number of traits needed to assess a horse’s auction value from 37 to just 8 key characteristics, making the process more straightforward and objective. The findings show that focusing on these high-impact traits can help breeders improve the quality and market value of horses, while buyers can use this information to make more informed purchases. This approach could also lead to the development of new economic genetic evaluation models and bring greater transparency to the equine market, benefiting the wider industry by supporting economic growth.

## 1. Introduction

The economic viability of sport horse production is intrinsically linked to sales prices [1,2]. In Ireland, the breeding and production of sport horses plays a significant role in the agricultural sector, with the equine industry contributing an estimated €816 million annually to the Irish economy [3]. The country’s reputation in sport horse breeding is highlighted by the success of the Irish Sport Horse (ISH) studbook, which ranked first in the World Breeding Federation for Sport Horses (WBFSH), for 24 out of the past 30 years based on competition results [4]. This underscores Ireland’s pre-eminence in producing high-quality event horses.

Conformation comprises many individual traits that are almost impossible to judge without objective measurements [5]. However, the development of linear profiling, which describes the horse for a range of traits on a linear scale from one biological extreme to the other, increases the objectivity and quality of phenotype recording [6]. Linear trait scoring has been used in livestock breeding since the 1970s to objectively assess phenotypic traits in relation to auction sale prices returns. Body weight was identified as a useful predictor of profitability in beef outputs from dairy farm production systems [7]. Muscular, skeletal, and functional linear type traits were associated with both live-weight and enhanced price of beef cattle [8]. In a study based on the Canadian Pro$ economic selection index, produced by Lactanet (Guelph, Canada), three traits—heel depth, body depth, and dairy capacity—demonstrated significant linear association with price in dairy cows, while bone quality was not significant [9].

The impact of conformation traits on the value and marketability of horses have been considered in previous thoroughbred and sport horse studies. In thoroughbred yearling’s, conformation, vet reports, and the pedigree rating metrics were all significant determinants of auction prices [10]. Also, in thoroughbred two-year olds, individual horse characteristics and pedigree quality variables were price determinants [11]. However mild conformational deviations of the carpus and fetlock in the forelimb were not found to affect thoroughbred racing performance when compared to horses without such deviations [12]. Conformation and Limb alignments were identified being important in sport performance and for the horse’s general health and wellbeing in Menorca Purebred Horses [13]. In online auctions, stock-type horses listed with videos received higher prices. Videos provided buyers with more information on horse’s conformation, suitability, and level of training [14]. Furthermore, in Ireland, Teagasc identified that the market value of sport horses is influenced by conformation, athleticism, breeding history, and performance potential. Horses destined for professional competition, such as showjumping and eventing, command higher prices due to their specific conformation and movement traits [15]. Previous studies have highlighted overall qualitative conformation judgements without specifically identifying which specific conformation or movement traits are influential as price determinants, this study addresses this critical literature gap by examining the relationship between objectively measured phenotypic traits in young event horses and their auction prices in Ireland. Recent empirical work from a range of countries confirms that auction markets continue to place measurable premiums on clearly defined genetic, performance, and management attributes. Analyses of online auctions for North-American stock-type horses reported significant price responses to age, sex, and colour, while training discipline shifted valuations by up to 18% [14]. A large-scale hedonic study of thoroughbreds re-entering sport careers showed that bidders rewarded chestnut and grey geldings aged five to six, especially when they already held sport-governing-body registrations, with average premiums of US$1000–1500 [16]. In Brazil, a 2024 evaluation of 452 Mangalarga Marchador lots demonstrated that auction form (online vs. live), coat colour, and maternal grandsire each altered mean price by 4–48%, highlighting the role of breed-specific aesthetic and pedigree cues in Latin-American markets [17]. Most recently, a UK veterinary-economics model of 1506 yearlings identified sire covering fee, consignment size, and catalogue timing as the strongest contemporaneous predictors of sale price, proving that buyer behaviour can be modelled robustly even under volatile economic conditions [18]. Collectively, these contemporary studies validate the current paper’s focus on quantifying objective price drivers and situate the Irish event-horse market within a broader, rapidly evolving international research context.

Despite its importance, no previous research has investigated how specific phenotypic traits influence sport horse sale prices. This study aimed to identify key conformation, movement, and athleticism traits that influence the sales price of young sport horses at public auctions in Ireland, with the goal of supporting more informed decision-making by breeders and buyers.

## 2. Materials and Methods

Anonymous public auction sales data, from 307 potential events horses sold in 2022–2023, were collected with owner consent at two Irish equine auction sales venues: Goresbridge sales Co. Kilkenny and Monart sales, Co. Wexford. These were the only companies that held specific auctions for potential event horses in Ireland during this period. Therefore, the data represents 100% of the available information for the two-year period. Data included age, sex, year, month of sale, sale date, venue identification, horse name, colour, height, sale price, and horse registration number. There were 278 three-year-old horses, 22 four-year-olds and 7 five-year olds. The dataset comprised geldings (*n* = 226) and female (81) horses. Most horses sold were Bay (*n* = 223) in colour, followed by Chestnut (*n* = 39), Grey (*n* = 37), and 8 other coloured horses. Most horses (*n* = 232) were between 164 cm and 172 cm in height, while 75 of the horses were taller than 175 cm (Appendix A: Table A1).

The horses were pre-selected for these auctions by a panel of auction selectors, who determined that they were suitable as potential event horses. This assessment took the form of a visual inspection of the horse standing, walking in hand, and moving in trot and canter in an enclosed arena. The horses were also assessed loose jumping or jumping under saddle, depending on their age. A full set of X-rays were also submitted to the sales companies in advance of the sale for inspection by potential customers. The Linear profile assessment for this study took place on the public viewing days of the sales, it was completed by a trained and experienced member of the ISH studbook stallion inspection panel. All horses were assessed by the one assessor. Each horse completed the same process, and all completed six, progressively challenging jumping efforts through the jumping lane. All jumping efforts of the horse were viewed prior to scoring on the linear profile for athleticism and attitude.

### 2.1. Linear Profile Traits

The ISH Studbook adopted the Netherlands Royal Dutch Warmblood Studbook (KWPN) linear profile system in 2008 for the Irish Sport Horse breed [19]. The phenotype describes the observable expression of an individual’s genotype [20]. The ISH linear profile scheme examines 37 phenotype traits, comprising 19 conformation, 9 movement, 8 jumping, and 1 temperament trait “Attitude” to describe the horse. Each trait is scored on a nine-point-scale of ‘A–I’, where ‘E’ is the centre score and ‘A’ and ‘I’ correspond to the two biological extremes. Table 1 shows the descriptive values for the phenotypic variables and illustrates descriptions of the biological extremes, scoring ranges, optimum scores, mean scores, standard deviations, and the significance level (*p*-value) of each trait in determining price at auction. The auction price (hereafter referred to as “price”) is measured as the hammer price, i.e., the actual final bid accepted at auction in euros (€), reflecting the sum paid for each horse, excluding any additional charges or fees. The *p* value in Table 1 indicates how statistically significant the association is between each individual conformation trait and the sales price of sport horses, while the optimum score identifies the value of each trait that has the strongest influence on maximising sales price.

Table 2, Table 3 and Table 4 provide the frequency distribution of the score range for Conformation (Table 2), Movement (Table 3), and Athleticism and Temperament (Table 4) traits, respectively, in young event horse auction sales in Ireland in 2022–2023. The complete dataset represents a thorough examination of 307 horses, with no missing observations. It provides a robust foundation for statistical analysis of linear factors influencing young event horse valuations in the Irish auction market.

### 2.2. Statistical Analysis

The structure of data and the distribution of the linear scores were analysed using the procedures FREQ and MEANS of SAS version 9.4 (SAS Institute Inc., Cary, NC, USA) (Table 1). To examine the relationship between linear profile trait scores and auction sales price of young event horses in Ireland three complementary analytical approaches were employed: mixed regression analysis, binary optimisation analysis, and PCA. Data on sales price were extracted directly from official auction records, ensuring accuracy and standardisation across all transactions included in this analysis.

#### 2.2.1. Determination of Trait Significance and Optimum Scores

The determination of optimum scores for each phenotypic trait was crucial to understand the biological direction of importance to sales price for each trait. Analysis using General Linear Models (GLM) procedure in SAS (SAS Institute Inc., 9.4) was performed. The procedural code specified class variables (sex, year, age, and all phenotypic traits). Each linearly scored trait, in total 37 traits, was analysed separately with the following linear model:

$$Y_{ij}\;=\;\mu \;+\;\alpha_{i}\;+\varepsilon_{j}$$

where $Y_{ij}$ is the dependent variable being tested (e.g., Head and Neck Connection), *µ* is the overall mean, $\alpha_{i}$ is the fixed effect of the independent variable Price and $\varepsilon_{j}$ is the random residual error. The solution option provided parameter estimates for each score level within each trait, enabling identification of the score with the strongest positive association with price. To validate the potential optimum scores, a subsequent Least Square Means (LSMEANS) analysis was performed. This dual approach ensured that optimum scores reflected both statistical significance (via parameter estimates) and economic magnitude (via adjusted means). The score level yielding the highest positive LSMean value was designated as the optimum for each trait (Table 1). This methodical process provided the foundation for the subsequent binary optimisation model (Model 2) by establishing biologically and statistically justified optimum scores for each trait.

#### 2.2.2. Multicollinearity Assessment and Variable Selection

Following the identification of significant traits and their optimum scores, an assessment of multicollinearity was conducted among the potential 29 traits demonstrating statistical significance, using Regression Procedure (PROC REG) with the Variance Inflation Factor (VIF) option. The analysis revealed levels of multicollinearity between Elasticity depicted a VIF of 3.03 and Scope (VIF of 4.22). Given the higher statistical significance of Scope for price prediction (adjusted R^2^ of 0.48 compared to 0.43 for Elasticity), Scope was retained, and Elasticity was removed from the model, which reduced Scope’s VIF to 3.6, bringing all VIF values well within acceptable thresholds (<5). The VIF threshold of five was selected based on established statistical literature suggesting that values below this threshold indicate acceptable levels of multicollinearity that do not substantially bias parameter estimates or standard errors [21].

#### 2.2.3. Model 1—All Recorded Individual Trait Scores Utilised

Variables were selected for inclusion in models by comparing *p*-values in the univariate analysis and based on their biological relevance. Then two mixed models were developed using a manual stepwise model-building procedure to identify the most important predictive variables associated with sales price according to the Akaike information criterion (AIC). The mixed-effects model was fitted using Restricted Maximum Likelihood (REML) estimation to analyse the relationship between phenotypic traits and auction sales price (in Euros). Model 1 using all scores (*a* to *i*) for each trait is described as follows:

$$Y_{ij}=\;\mu +\sum_{k=1}^{P}\beta_{k\;}X_{ijk}\;+\;a_{i}+\;{yr}_{j}+\epsilon_{ij}$$

where $Y_{ij}$ is the auction price in Euros for observation in year *j* for animal I; *μ* is the overall mean; $\beta_{k\;}$ are the fixed effects coefficients for the 17 retained phenotypic liner traits Head Neck Connection, Length of Neck, Position of Shoulder, Length of Croup, Quality of Legs, Walk Trot and Canter traits, Take off Technique, Scope, Care, and Attitude traits; $X_{ijk}$ are the phenotypic trait scores; $a_{i}$ is the random effect of animal ID; ${yr}_{j}$ is the random effect of year; and $\epsilon_{ij}$ is the residual error term. Non-significant traits for conformation were, body shape, body direction, Length of Neck, Position of Neck, Muscling of Neck, Height of Withers, Line of Back, Line of Lions, Shape of Croup, Stance of Forelegs, Stance of Hindlegs, Stance of Pastern, Shape of Feet, Heels, and Substance of Legs. For movement, they were Trot Length of Stride, Trot Impulsion, Canter Balance; For Athleticism, Technique of Forelegs, and Elasticity. The temperament trait of Attitude was also not significant in the model.

#### 2.2.4. Optimum Score Linear Trait Model

A second similar model, Model 2 was developed to enhance model parsimony by utilising optimum trait scores (OPT), detailed above, rather than the full *a* to *i* (1–9) scale for each trait. Each phenotypic trait was scored as a binary variable (1 or 2). The optimum score represented the specific value within the linear scoring range that demonstrated the strongest positive association with sales price for each trait. Following manual stepwise building procedure the 17 traits retained in the model were, Head and Neck connection, Length of Neck, Position of Shoulder, Length of Croup, Quality of Legs, Walk Length of Stride, Trot Elasticity and Balance, Canter Length of Stride and Impulsion, Take off Direction, Take off Quickness, Technique of Back and Technique of Haunches, Scope and Care. Non-significant traits for Model 2 were the same as detailed above for Model 1.

The transformation of linear profile scores into binary variables confers several methodological and practical benefits, particularly evident in the context and aims of this research. This approach sharply enhances the interpretability of the regression model. This contrasts with models based upon multiple category levels, where interpretation can be ambiguous and coefficients for intermediate scores are less meaningful for practical decision-making. Secondly, deriving binary variables according to statistically defined optimum scores, as informed by adjusted means and fixed-effect estimates, directly supports application in selection indexes and genomic breeding programmes. Studbooks and breeders can be guided to target, promote, and select for those trait expressions empirically linked with higher market valuations, thereby aligning selection objectives more closely with economic returns. Additionally, binary transformation strengthens model parsimony and robustness. It mitigates the risk of overfitting—often a hazard with models incorporating numerous ordinal levels—and reduces standard errors, yielding more stable parameter estimates even when sample sizes for each raw category are limited. In summary, adopting a binary scoring methodology does not merely simplify analysis—it tailors the model outputs to practical breeding, sales, and industry decision-making. It clearly identifies which optimal trait expressions have the biggest impact on price, facilitating targeted genetic improvement, more transparent market communication, and streamlined evaluation for all industry stakeholders.

#### 2.2.5. Principal Component Analysis (PCA)

**Figure 1:**
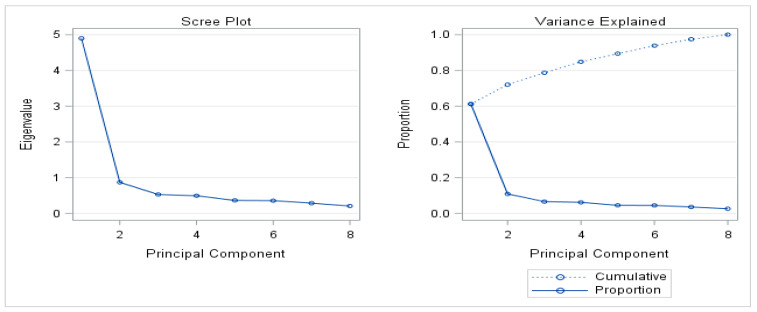
Biplot of Principle Components (PC) 1 to 7 calculated 8 Phenotypic traits of event horses sold at public auction 2022 and 2023.

Finally, PCA was conducted on the dataset to transform the original variables into a new set of orthogonal variables, known as principal components. In previous research on Belgium warmbloods principal component analysis was suggested to reduce the number of Linear Profile traits required and so aid breeders understanding [22]. The initial Principal Component Analysis (PCA) in this study was conducted on all 37 linear traits to identify the most informative features. Prior to performing PCA, the raw linear trait data were auto-scaled by centring each variable to a mean of zero and standardising to unit variance (standard deviation of one); this ensured all variables contribute equally to the analysis, removing effects of differing scales and means. This was achieved in SAS by employing the proc standard procedure with the options mean = 0 std = 1 on the selected variables. Following standardisation, PCA was performed using the proc princomp procedure on the standardised dataset. The top eight principal components were selected based on their eigenvalues. A further principal component analysis (PCA) was conducted to evaluate the contribution of eight phenotypic traits—Scope, Elasticity, Canter Impulsion, Canter Balance, Technique of the Back, Canter Length of Stride, Trot Balance, and Trot Impulsion—to the variation in the data. The extraction and visualisation of results through a biplot is shown in Figure 1. The first four principal components were retained based on eigenvalues and variance explained, highlighting the most influential traits.

The final PCA can be represented by the following equation:

$$\begin{array}{l} \mathrm{PC1}=\beta 1\;\mathrm{(Scope)}+\beta 2\;(\mathrm{Elasticity})+\beta 3\;(\mathrm{Canter}\mathrm{Impulsion})+\beta 4\;(\mathrm{Canter}\mathrm{Balance})\\ +\beta 5\;(\mathrm{Technique}\mathrm{of}\mathrm{Back})+\beta 6\;(\mathrm{Canter}\mathrm{Length}\mathrm{of}\mathrm{Stride})+\beta 7\;(\mathrm{Trot}\mathrm{Balance})\\ +\beta 8\;(\mathrm{Trot}\mathrm{Impulsion}) \end{array}$$

## 3. Results

After significance testing eight traits were found not to significantly impact sales price and so Body Direction, Muscling of Neck, Line of Lions, Stance of Forelegs, Stance of Hindlegs, Shape of feet, Heels, and Walk Correctness were removed from further analysis. Elasticity was also removed from the analysis due to its high multicollinearity.

### 3.1. Model 1

Model 1, using all recorded linear scores (*a* to *i*) for each trait demonstrated good convergence, reaching criterion after seven iterations with a final −2 Res Log Likelihood of 4430.74. The model fit statistics indicated reasonable performance with AIC = 4436.70. However, due to nine possible scores for each trait, the results derived from the first model were limited and are therefore included for reference only (Appendix A: Table A2). While not useful in determining the relevant biological direction of a trait, the model did identify several traits that significantly influenced sales price. The highly significant traits (*p* < 0.001) were Head and Neck Connection, Scope and Care over the fence. Significant traits (*p* < 0.05) identified were Walk Length of Stride, Trot Elasticity, Trot Balance and Take off Direction.

### 3.2. Model 2

The optimised Model 2 full results are reported in Table 5. They identify several traits are highly significantly associated with higher sales prices. Horses with a light Head and Neck Connection (optimum score: 3) had an estimated price increase of €4814, while those with lean Quality of Legs (optimum score: 4) showed an increase of €4445. A long Walk Length of Stride (optimum score: 3) corresponded to a €2991 increase, and considerable Scope (optimum score: 2) was linked to the highest estimated price increase of €8445. Further, a long Length of Croup (score 3) was associated with an estimated price increase of €2815. Interestingly, a very elastic Trot (score 2) was associated with a significant decrease in price (€−7451), whereas a balanced Trot when carrying (score 2) resulted in an estimated increase of €3545. An upward Take-off Direction (score 2) also contributed positively (€1785).

While eight additional traits did not individually reach statistical significance, their inclusion improved the overall model fit and they were therefore retained. Following identification of optimum trait scores and their impact in Model 2, we conducted a principal component analysis (PCA) to better understand the underlying data structure and explore dimensionality reduction.

### 3.3. PCA Results

Table 6 shows the results of the PCA analysis. The first principal component (PC1) accounted for 61.19% of the variance, with high loadings across all traits, particularly scope (0.38), Canter Impulsion (0.37), Elasticity (0.36) and Canter Balance (0.36) had the highest loadings, with the remaining four traits, technique of back, Canter Length of Stride, Trot Balance and impulsion having the same loading score (0.34). This indicates that PC1 represents a general athleticism factor. The second principal component (PC2) explained an additional 10.85% of the variance, with strong contributions from trot impulsion (0.49) and trot balance (0.46), highlighting traits related to trot dynamics. PC3 contributed 6.61% of the variance and was characterised by high loading for Canter Length of Stride (0.82). PC4 (6.16%) identified a high loading for Technique of the Back (0.53). The subsequent PCs explained progressively smaller proportions of variance: PC5 (4.55%), PC6 (4.46%), and PC7 (3.58%).

The cumulative variance explained by the first four PCs was 84.82%, while all seven PCs together accounted for 97.4%, indicating that these traits collectively provide a comprehensive explanation of variability in the data. In summary, reducing the number of traits from 37 to 8 allowed us to focus on key phenotypic characteristics that explain a sizable portion of variability among the linear profile traits of the horses at these sales.

The scree plot was used to determine the optimal number of principal components to retain, it indicated that the first four components captured the most significant amount (84.82%) of variance (Figure 1).

Table 7 below provides a summary of the traits most strongly associated with each principal component.

A summary of the significant phenotypic traits influencing auction sales prices of young event horses identified using two analytical models: 1. Full linear trait scores, 2. Binary optimum scores, and the traits identifying the most variance in the data through PCA are presented below (Table 8).

## 4. Discussion

The complex nature of eventing, encompassing three distinct disciplines; dressage, show jumping, and cross-country, creates unique challenges in identifying optimal trait combinations that influence market value. International level events are star rated and commence with CCI1* and progress to CCI5* in which the long format competition takes place over three or more days, these competitions are overseen by the Federation Equestre Internationale (FEI). The FEI General Regulations and Eventing Rules differentiate five-star eventing (CCI5*-L) from other levels based on difficulty, technical requirements, and course specifications [23]. Dressage at five-star level requires the most advanced dressage movements, such as half-pass, multiple flying changes and each horse is judged by multiple judges. The cross-country phase then presents a diametrically opposed challenge: horses must negotiate 30–45 fixed obstacles over distance (up to 6840 m), on undulating terrain with maximum obstacle height (1.20 m) and drops (up to 2 m), requiring explosive power, cardiovascular endurance, and split-second decision-making. Other studies have shown that five-star courses test horses to physiological limits, with heart rates sustaining >200 bpm for 11–12 min [24]. Finally, the horses must recover overnight to meet the requirements of the trot up and veterinary check prior to the showjumping phase. The showjumping phase provides a challenging course of 12–16 obstacles with a maximum fence height of 1.30 m to test precision, suppleness and carefulness of the horse after the rigours of the cross-country phase. Prior to entering eventing competitions at an international level, both horses and riders must meet Minimum Eligibility Requirement (MER) to ensure that they are competent and fit enough to complete at each successive level of eventing.

International level event horses must reconcile seemingly contradictory traits, therefore understanding the economic value of linear profile phenotypic traits is important to enhancing the efficiency of breeding programmes, improve the quality of sport horses, and ultimately influence their market prices. This research contributes to the ongoing effort to refine and optimise the use of linear profiling in the equine industry in assessing conformation traits [22] and ensuring that breeding goals align with market demands and economic outcomes for breeders [13]. Linear assessment trait evaluation system was identified as being useful as a quantitative selection criterion in the breeding of Thoroughbred horses in 1996 [25,26]. Linear profiling was recognised as a useful tool for studbooks and equine research but there were initial challenges with its use, and the uptake of the methodology was slow [17,26]. Most early research focused on determining how young sport horses align with the breeding objective of studbooks [27,28] and in determining the heritability of various traits for potential use in genetic estimation models [29,30]. More recently the linear trait research has focused on how its use can contribute to the eradication of major conformation defects (specifically hock and knee) which make some Pura Raza Española (PRE) sport horses unsuitable for sport [13]. A further study of Menorca Purebred horses identified strong heritability’s for undesirable traits such as splay-footed forelimb, closed hocks, camped under, pigeon-toed forelimb and coon foot using linear profiling. The study concluded that selection efforts should focus on identifying more prevalent defects with higher heritability for inclusion in the breeding programme [13].

Increasingly, linear profile research focused on predicting performance in the disciplines of showjumping and dressage [31,32], and specifically dressage performance [33,34] and genetic heritability’s for potential economic trait assessment [35].

Borowska and Lewczuk conducted a comparative analysis of traditional 100-point judging systems and linear profiling to identify traits of specific importance in dressage and jumping horses [32]. Warmblood mares varied in a few linear conformation traits, most significantly in the position of the shoulder, line of the loins and/or shape of the croup. The analyses of linear movement traits illustrated that dressage mares moved better in walking and more balanced canter than jumping ones, differences that were not evident in the overall 100-point system [32]. The research underscores the efficacy of linear profiling in providing more nuanced, objective information on conformation and movement traits compared to traditional point methods [22]. Furthermore, it corroborates the association between certain traits and discipline-specific performance-enhancing attributes.

This study’s findings provide unprecedented insights into traits of importance for sales price in young event horses through complementary analytical approaches. The convergence of results across these distinct methods offers robust evidence for key traits influencing auction prices, with Scope emerging as the most significant factor. The PCA revealed a set of 8 traits out of a total of 37 linear traits that collectively explain the vast majority of variance in the dataset. These traits are as follows: Trot Impulsion, Trot Balance, Canter Length of Stride, Canter Impulsion, Canter Balance, Technique of the Back, Scope and Elasticity.

The optimised regression analyses identified conformation traits of Head and neck connection, Length of croup, Quality of Legs, movement traits of Walk length of Stride, Trot Elasticity and Balance, Take off Direction and Scope as extremely significant predictors of sales price. Among these, Scope Head–neck Connection, Quality of Legs and trot balance stood out particularly as positively affecting the price of the horse. The linearly scored traits that significantly affected sales price in event horses demonstrated a biological expression towards one of the extremes. Thus, horses scoring towards “a” on the linear assessment scale, the higher the probability of that the horse achieved a better sales price. This is similar to findings in a recent Swedish study that found horses scoring towards “a” in the linear scale for; direction and power of take-off, balance, scope, and technique of the back, demonstrated a highly significant (*p* < 0.0001) probability of those horses competing on a high level in show jumping [33].

These results not only confirm the importance of certain traditionally valued traits but also highlight the nuanced interplay of various physical, movement and athleticism characteristics in determining a young event horse’s market value.

### 4.1. Athleticism and Jumping Capacity

A clear emphasis on dynamic performance traits was identified in this study. Scope was identified as a crucial determinant of sales price, with horses showing much scope (score 2) achieving an estimate of €8445 over those horses with less scope. The PCA loadings were heavily influenced by Scope (loading = 0.38), Elasticity (loading = 0.36), Canter Balance (loading = 0.36) and Canter Impulsion (loading = 0.37), suggesting that these components represent a combination of factors related to jumping ability and movement quality. Specifically, Scope contributed significantly to PC1, which explained over 61% of the variance, reinforcing its importance in disciplines like showjumping where scope, back strength and flexibility are critical for clearing obstacles. This aligns with the observation that rapid power bursts in middle gluteal muscles during approach and take-off are crucial performance indicators and represent objective indicators of a horse’s capacity for executing larger fences or its ‘scope’ [36]. These findings align with previous research that emphasises the importance of scope and canter quality for jumping horses [37]. That research described difference between trot and canter, particularly noting how the asymmetrical nature of the canter means that flexion and extension of the vertebral column, contribute to the changes in stride length [37]. This description usefully draws a strong link between head and neck connection, the vertebral column and the quality and length of stride in the canter, which may provide a causal link to the extreme significance of the head and neck connection conformation trait found in both regression analysis to the price received for a horse. Direction and power of take-off, balance, scope, and technique of the back were jumping traits that had a highly significant (*p* < 0.0001) association with show jumping performance in a study on Swedish warmbloods [35]. These results are also agreement with findings in a study by Stock et al. in 2022 [38], where the strongest genetic trends in Oldenburg jumping horses were observed for power of take-off and jumping ability.

Take off Direction (€1785) was a significant athleticism trait in the regression analysis which positively impacted sales price where horses scored the optimum score of 2 (upwards). The importance of this trait aligns with previous findings of jumping traits in stallion inspections which highlighted that ‘take-off’ had very strong genetic correlations with subsequent performance in international show-jumping competitions [39]. The generation of engagement, impulsion and hindlimb muscle power, observed on take-off, when selecting jumping horses and was identified as an objective performance indicators for the sport of showjumping [36].

The regression analysis (Model 1) highlighted Care, carefulness over the fence, as a significant trait. However, this significance was not maintained in the optimised model (Model 2, *p* = 0.2073) or reflected in the PCA components. While care may be important for show jumping competition performance, our analysis suggests it may not be a primary driver of young event horse prices at auction. This could indicate that buyers prioritise other athletic traits such as Scope and Take-off Direction when purchasing young event horses. Neither technique of the fore limb or haunches were found to be significant in this study. This aligns with a study on muscle function and kinematics in jumping which identified that less emphasis is placed on the forelimb during the approach stride and forelimb and hindlimb joint articulation during flight, which the study found did not differentiate jumping performance [36].

### 4.2. Movement Quality and Dynamic Performance

Thoroughbred studies have established a correlation between walk stride length, enhanced sales price and superior galloping ability, a crucial attribute, not only for thoroughbreds but for top-tier event horses [11]. This sale price study corroborates the Thoroughbred study findings, identifying a significant positive price estimate of €2991 (*p* < 0.001) associated with walk length of stride. It is worth noting that horses with shorter strides have been found to be more susceptible to front leg lameness, as the increased frequency of ground contact places greater pressure on the limbs [40]. The optimum score in this study was 3, indicating obviously long walk stride length.

The results indicate that purchasers value the trot in young horses as a potential indicator of future competition success. This aligns with research that found that trot characteristics were among the most important criteria for selecting dressage horses [30]. The emphasis on trot quality suggests that buyers recognised these traits as foundational for developing more advanced movements required in higher-level dressage competitions. This is consistent with findings by [28,41], who reported that movement quality, especially in trot, was a key factor in predicting future sport performance in young horses. This was supported by Back et al. [42], who determined that kinematic patterns in young horses are relatively stable and can be used to predict potential adult performance. The trot’s importance likely stems from its role as a fundamental gait for developing collection, extension, and lateral movements all crucial elements in dressage tests. Both balance and impulsion during the trot are crucial for dressage horses, especially when executing collected movements [43]. Thus, purchasers’ focus on trot characteristics in young horses appears to be a strategic approach to identifying individuals with the potential for success in the dressage element of future competitions.

### 4.3. Conformation Factors That Impact Price

The regression analysis identified traits such as Head–neck Connection (€4813) and Walk Length of stride (€2991) as highly significant (*p* < 0.001), indicating a strong association with sales prices of young event horses. The importance of head–neck connection in relation to balance and coordination in sport horses has been previously emphasised [44] and in their locomotion patterns [43,44]. In a study of event riders’ preferences for young event horse conformation in Ireland, 50% considered a light head and neck connection important [45]. Irish horses in the studied population align well with this ideal of tending towards a light head and neck connection, with those scoring an optimum score of 3 (obviously light) achieving a highly significant price premium of €4813 over those with average or obviously heavy Head and Neck connections [5].

Holmstrom [43] indicated that flatter croup inclination was prevalent in groups of elite performance horses in dressage and showjumping. However, the relevance of these findings to the present study is unclear due to differences in reference points used to determine croup conformation. Interestingly, elite riders in the event horse conformation study expressed a preference for horses trending towards a more sloping croup shape, with only 5.6% of horses presented in that young horse competition study having flat croups [45]. It is noteworthy that the present study found the length of the croup, rather than its slope, to be highly significant in affecting sales price. The trait of “Slope of the croup” was not significant in any of the analysis in this study (Table 7). The optimum score for croup length was 3, which is outside the average scores (4, 5, 6) and equates to an obviously long croup. Such horses achieved a price premium of €2815, all else being equal (Table 5). This finding aligns with recent research on muscle function during jumping, which identified hindlimb muscle power as an important factor when selecting jumping horses and as a performance indicator for equine jumping [36]. While this study does not directly link hindquarter and hindlimb muscle power to a longer croup, it is logical to infer that a longer, and therefore larger, croup provides greater potential for enhanced muscle mass. This area presents an intriguing avenue for future research.

### 4.4. Non-Significant Traits

Conformation traits traditionally regarded as critical in assessments such as body direction, length, position and muscling of neck, height of withers, line of back and loins, stance of forelegs and hindlegs, stance of pastern, shape of feet, heels, and quality and substance of legs did not emerge as significant predictors in the regression analysis (Table 5). Many of these traits have historically been considered important by breeders and evaluators, yet they did not exhibit a strong association with sales price in this study. Walk correctness has been consistently identified as an important trait in sport horse performance, particularly for disciplines like dressage where gait regularity and precision are critical. Studies by Holmstrom et al. [43] and Clayton [37] provide strong evidence that walk correctness is a key factor influencing performance outcomes. In purchasing potential event horses, 100% of four star event riders identified walk correctness as essential, when interviewed [45]. However, in a recent study traits describing aspects of walk were not significant for either show jumping or dressage performance [33].

Several factors may explain the lack of significance of these traits. The most obvious is the pre-selection process at auctions, where horses with poor conformation may have already been excluded, leaving a more uniform population in terms of these traits. The significance of these traits may vary depending on the specific population or context, such as stallion selections or different breeding programmes requiring more comprehensive trait selection identified through heritability estimates rather than sale value considerations. A study of ISH buyers revealed a significant discrepancy between the traits prioritised by potential purchasers and those deemed important by producers, such as gender, colour, and pedigree [46]. Modern breeding techniques may also be evolving and therefore influencing the relevance of certain traditional conformation traits. The possibility of reducing the number of strongly correlated linearly scored traits assessed in horses was proposed by Samoré [47]. In a very recent study which investigated the interrelationship between the linearly scored traits in Swedish Warmbloods (SWB) through factor analysis and the estimated genetic parameters for the resulting underlying factors through multiple-trait analyses [35]. That study effectively reduced the Linear traits used from the standard 59 in SWB to 14 to be included in multiple-trait genetic evaluation or in genomic analysis for warmblood horses. In attempting to reduce the number of traits for analysis and ease of understanding by breeders, care should be taken to ensure the nuances of interplay between traits is not lost. However, our study clearly indicates that the market is influenced by fewer than the standard 37 linear profile traits and may be shifting towards valuing performance-related traits over traditional conformation attributes.

### 4.5. Limitations

In addition to the acknowledged subjectivity inherent in linear trait assessment and market context variations, additional methodological limitations warrant consideration. Pre-sale selection bias represents a factor that may influence the generalisability of these findings. Vendors typically make strategic decisions about which horses to consign to auction based on anticipated market reception, reserve prices, and perceived commercial value. This selective process means that the horses appearing at auction may not represent the full spectrum of the breeding population but rather a curated subset deemed commercially viable. The relationships between phenotypic traits and prices observed in this study may reflect both genuine market preferences and the pre-existing bias introduced by vendor selection strategies.

Assessor bias constitutes another potential source of variation that could influence the validity of linear trait scoring. Recent research has demonstrated biases in equine evaluation, with assessors showing variability in scoring consistency and potential influences from factors such as regional preferences, and prior expectations [48]. While standardised scoring protocols and the consistent use of one assessor in this study attempt to mitigate such effects, the inherent subjectivity of morphological assessment must be considered as a limitation. Finally, as the Irish Sport Horse population include and phenotypic subset within the broader equine population. The genetic patterns, selection pressures, and breeding objectives unique to Irish Sport Horses may limit the transferability of these findings to other populations.

### 4.6. Future Application and Genetic Implications

The applicability of these findings extends beyond the immediate Irish event horse population and offers valuable insight for other breeds and other equestrian disciplines (i.e., Dressage or showjumping sales) and markets internationally. While the strength and direction of associations between phenotypic traits and auction prices may vary depending on breed-specific selection objectives, sporting disciplines, genetic backgrounds, and cultural preferences, the methodological approach and core results have broader relevance. The identification of traits with substantial commercial impact can inform breeding programmes in diverse contexts, supporting the development of selection indices tailored to local or breed-specific performance goals. Furthermore, by demonstrating that linear trait assessment reliably predicts market value, this research encourages the harmonisation of phenotyping protocols and pricing analyses across countries, which could facilitate international benchmarking and data-sharing initiatives. As such, these insights provide a foundation for comparative studies and collaborative genetic evaluations, ultimately enhancing the efficiency and transparency of sport horse selection and marketing on a global scale.

The phenotypic associations identified in this study provide a valuable foundation for enhancing breeding programmes through integration into genetic evaluation systems. Detailed linear trait data can improve the accuracy of estimated breeding values (EBVs), particularly for young horses lacking performance records, facilitating earlier and more informed selection decisions. Moreover, incorporating these traits into multi-trait selection indices enables balanced breeding strategies that optimise both morphological and performance characteristics. Advances in equine genomics now allow the combination of high-quality phenotypic data with genomic information to increase prediction accuracy and genetic gain. Harmonising linear trait recording protocols across populations would further support international genetic evaluations, enabling direct comparisons of EBVs and enhancing global breeding efforts. Overall, these findings offer promising avenues to accelerate genetic progress and optimise event horse breeding through the systematic use of phenotypic and genomic data.

## 5. Conclusions

This study provides valuable insights into the key phenotypic traits influencing the sales price of young event horses in Ireland, with important implications for breeders, studbooks, and the wider equine policy makers. By employing optimised regression analysis, we were able to identify and reduce the number of traits from the original 37 traits to those that are most critical for assessing and predicting sales price, while maintaining a robust understanding of the factors that buyers prioritise. This analysis identified eight traits as predictors of positive sales price estimates, Head–neck connection, Quality of legs, Walk Length of Stride, Trot Balance and Scope, which were highly significant trait predictors of sales price (*p* < 0.001), while Length of Croup, Trot Elasticity, and Take-off Direction were significant in determining sales price. These eight traits clearly demonstrate the importance buyers place on specific conformation traits, balance, movement, and athletic ability when they are purchasing young event horses. The identification of these traits aligns with existing literature on further sport horse performance, further validating the robustness of our price impact findings.

The principal component analysis (PCA) identified eight phenotypic traits—Trot Impulsion, Trot Balance, Canter Length of Stride, Canter Impulsion, Canter Balance, Technique of the Back, Scope, and Elasticity—that when four PCs were retained, explained 84.82% of the variation in the linear profile traits of event horses sold at public auctions in 2022 and 2023. If seven principal components (PCs), were retained these traits which collectively explained 97.4% of the total variance of the dataset.

By focusing on traits like Head and Neck Connection, Trot and Canter Balance, Take-off direction, Scope, and Elasticity, breeders can enhance the marketability and performance potential of their horses. Furthermore, by reducing the number of traits assessed through linear profiling while maintaining predictive power it opens opportunities for its widespread use in assessing price determinants across various sectors of the equine industry. The identified traits could form the basis of a new economic index for event horse breeding, or a buyer’s index facilitating more accurate valuation and selection decisions. Such tools would enhance market efficiency and transparency. The methodology contributes to the ongoing refinement of linear profiling utilisation in the equine industry and provides a valuable foundation for enhancing breeding programmes through integration of the methodology into genetic evaluation systems. These findings have significant implications for breeders, studbook policy makers, and other stakeholders in the equine industry who aim to optimise horse selection and improve market outcomes.

## Figures and Tables

**Table 1:** Mean score, standard deviation (SD), range, significance level, and optimum sales price score for linearly scored phenotypic traits of young event horses sold at auction 2022–2023.

	Trait	Extreme Values	Score Range 1–9	Mean	SD	*p*-Value	Optimum Score
	**Conformation**						
**1**	Body Shape	rectangular–square	2 to 6	3.88	0.67	***	3
**2**	Body Direction	uphill–downhill	2 to 8	5.08	1.08	n.s.	8
**3**	Head and Neck Connection	light–Heavy	3 to 6	5.04	1.04	***	3
**4**	Length of Neck	long–short	3 to 8	5.07	0.87	***	3
**5**	Position of Neck	vertical–horizontal	2 to 8	4.71	0.99	***	3
**6**	Muscling of Neck	heavy–poor	3 to 7	4.65	0.74	n.s.	3
**7**	Height of Withers	high–low	2 to 7	4.17	0.81	**	3
**8**	Position of Shoulder	sloping–straight	3 to 8	4.74	1.29	**	7
**9**	Line of Back	roached–weak	3 to 7	5.16	0.86	**	3
**10**	Line of Loins	roached–weak	3 to 7	4.83	0.94	n.s.	3
**11**	Shape of Croup	sloping–straight	1 to 7	4.20	0.99	**	4
**12**	Length of Croup	long–short	3 to 8	4.98	1.08	**	3
**13**	Stance of Foreleg	over at knee/back at knee	4 to 7	5.35	0.63	n.s.	7
**14**	Stance of Hindlegs	sickle–straight	3 to 8	4.89	1.08	n.s.	4
**15**	Stance of Pastern	weak-upright	3 to 7	4.70	0.82	**	3
**16**	Shape of Feet	wide–narrow	2 to 7	4.29	0.84	n.s.	5
**17**	Heels	high–low	3 to 8	5.23	0.64	n.s.	5
**18**	Quality of Legs	lean–blurred	3 to 7	5.03	0.56	***	4
**19**	Substance of Legs	heavy–fine	4 to 7	5.07	0.58	*	7
	**Movement**						
**20**	Walk Length of Stride	long–short	3 to 7	4.32	0.77	**	3
**21**	Walk Correctness	toed in–toed out	2 to 7	4.95	0.78	n.s.	5
**22**	Trot Length of Stride	long–short	3 to 7	4.48	1.01	*	3
**23**	Trot Elasticity	elastic–stiff	2 to 8	4.55	1.04	*	2
**24**	Trot Impulsion	powerful–weak	2 to 8	4.67	1.02	***	2
**25**	Trot Balance	carrying–pushing	2 to 7	4.78	1.03	***	2
**26**	Canter Length of Stride	long–short	3 to 7	4.46	0.88	***	3
**27**	Canter Impulsion	powerful–weak	2 to 8	4.70	1.03	***	2
**28**	Canter Balance	carrying–pushing	2 to 8	4.97	1.13	***	2
	**Athleticism**						
**29**	Take off Direction	upwards–forwards	2 to 8	4.87	1.48	***	2
**30**	Take off Quickness	quick–slow	2 to 8	4.80	1.23	***	3
**31**	Technique Forelegs	bent–stretched	2 to 7	4.36	1.28	***	3
**32**	Technique Back	rounded–hollow	2 to 8	4.88	1.32	***	2
**33**	Technique Haunches	open–tight	2 to 8	4.63	1.36	***	2
**34**	Scope	much–little	2 to 8	4.49	1.23	***	2
**35**	Elasticity	supple–stiff	2 to 8	4.70	1.26	***	2
**36**	Care	careful–not	2 to 7	3.58	0.97	***	2
**37**	Attitude	much–little	2 to 6	3.16	0.75	***	2

*p*-Value, * *p* < 0.05, ** *p* < 0.01, *** *p* < 0.001, n.s., not significant.

**Table 2:** Frequency distribution of conformation trait variables in young event horse auction sales in Ireland in 2022–2023.

Score Freq	Trait Score Distribution							Trait Score Distribution						
Trait	Body Shape							Body Direction						
Score	b	c	d	e	f			b	c	d	e	f	g	h
Freq. (%)	3 (1%)	73 (24%)	194 (63%)	31 (10%)	6 (2%)			1 (0%)	10 (3%)	101 (33%)	78 (25%)	87 (28%)	29 (9%)	1 (0%)
Trait	Head/Neck Connection							Length of Neck						
Score	c	d	e	f	g			r	c	d	e	f	g	r
Freq. (%)	17 (6%)	82 (27%)	105 (34%)	78 (25%)	24 (8%)			1 (0%)	8 (3%)	69 (22%)	137 (45%)	81 (26%)	11 (4%)	1 (0%)
Trait	Position of Neck							Muscling of Neck						
Score	c	d	e	f	g			c	d	e	f	g		
Freq. (%)	27 (9%)	119 (39%)	79 (25%)	80 (26%)	2 (1%)			8 (3%)	128 (42%)	139 (45%)	27 (9%)	5 (2%)		
Trait	Height of Withers							Position of Shoulder						
Score	b	c	d	e	f	g		c	d	e	f	g	h	
Freq. (%)	1 (0%)	54 (18%)	167 (54%)	64 (21%)	20 (7%)	1 (0%)		44 (14%)	136 (44%)	21 (7%)	69 (22%)	36 (12%)	1 (0%)	
Trait	Line of Back							Line of Lions						
Score	c	d	e	f	g			c	d	e	f	g		
Freq. (%)	2 (1%)	70 (23%)	130 (42%)	88 (29%)	17 (6%)			11 (4%)	124 (40%)	87 (28%)	77 (25%)	8 (3%)		
Trait	Shape of Croup							Length of Croup						
Score	a	b	c	d	e	f	g	c	d	e	f	g	h	
Freq. (%)	1 (0%)	1 (0%)	62 (20%)	167 (54%)	24 (8%)	51 (17%)	1 (0%)	12 (4%)	118 (38%)	69 (22%)	82 (27%)	25 (8%)	1 (0%)	
Trait	Stance of Forelegs							Stance of Hindlegs						
Score	d	e	f	g				c	d	e	f	g	h	
Freq. (%)	8 (3%)	201 (65%)	80 (26%)	18 (6%)				27 (9%)	96 (31%)	89 (29%)	76 (25%)	18 (6%)	1 (0%)	
Trait	Stance of Pastern							Shape of Feet						
Score	c	d	e	f	g			b	c	d	e	f	g	
Freq. (%)	32 (10%)	64 (21%)	177 (58%)	31 (10%)	3 (1%)			2 (1%)	46 (15%)	141 (46%)	103 (34%)	10 (3%)	5 (2%)	
Trait	Heels							Substance of Legs						
Score	c	d	e	f	g	h		d	e	f	g			
Freq. (%)	1 (0%)	23 (7%)	196 (64%)	80 (26%)	6 (2%)	1 (0%)		31 (10%)	235 (77%)	30 (10%)	11 (4%)			
Trait	Quality of Legs													
Score	c	d	e	f	g									
Freq. (%)	5 (2%)	23 (7%)	242 (79%)	31 (10%)	6 (2%)									

**Table 3:** Frequency distribution of movement trait variables in young event horse auction sales in Ireland in 2022–2023.

Score Freq	Trait Score Distribution							Trait Score Distribution				
Trait	Walk Correctness							Walk Length of Stride				
Score	b	c	d	e	f	g		c	d	e	f	g
Freq. (%)	1 (0%)	16 (5%)	38 (12%)	202 (66%)	41 (13%)	9 (3%)		24 (8%)	189 (62%)	71 (23%)	18 (6%)	5 (2%)
Trait	Trot Elasticity							Trot Length of Stride				
Score	b	c	d	e	f	g	h	c	d	e	f	g
Freq. (%)	2 (1%)	31 (10%)	141 (46%)	80 (26%)	38 (12%)	13 (4%)	2 (1%)	43 (14%)	138 (45%)	76 (25%)	37 (12%)	13 (4%)
Trait	Trot Impulsion							Trot Balance				
Score	b	c	d	e	f	g	h	b	c	d	e	f
Freq. (%)	2 (1%)	30 (10%)	111 (36%)	98 (32%)	58 (19%)	6 (2%)	2 (1%)	14 (4%)	28 (9%)	101 (33%)	98 (32%)	66 (21%)
Trait	Canter Impulsion							Canter Length of Stride				
Score	b	c	d	e	f	g	h	c	d	e	f	g
Freq. (%)	2 (1%)	27 (9%)	112 (36%)	105 (34%)	44 (14%)	16 (5%)	1 (0%)	37 (12%)	132 (43%)	102 (33%)	32 (10%)	4 (1%)
Trait	Canter Balance											
Score	b	c	d	e	f	g	h					
Freq. (%)	1 (0%)	27 (9%)	85 (28%)	90 (29%)	78 (25%)	24 (8%)	2 (1%)					

**Table 4:** Frequency distribution of athleticism and temperament (Attitude) trait variables in young event horse auction sales in Ireland in 2022–2023.

Score Freq	Trait Score Distribution							Trait Score Distribution					
Trait	Take off Direction							Take of Quickness					
Score	b	c	d	e	f	g	h	b	c	d	e	f	g
Freq. (%)	4 (1%)	58 (19%)	100 (33%)	8 (3%)	90 (29%)	42 (14%)	5 (2%)	4 (2%)	29 (9%)	135 (44%)	29 (9%)	83 (27%)	27 (9%)
Trait	Technique Back							Technique Forelegs					
Score	b	c	d	e	f	g	h	b	c	d	e	f	g
Freq. (%)	2 (1%)	40 (13%)	102 (33%)	60 (20%)	59 (19%)	39 (13%)	5 (2%)	9 (3%)	62 (20%)	144 (47%)	20 (7%)	45 (15%)	27 (9%)
Trait	Technique Haunches							Care					
Score	b	c	d	e	f	g	h	b	c	d	e	f	g
Freq. (%)	11 (4%)	47 (15%)	118 (38%)	33 (11%)	69 (22%)	26 (8%)	3 (1%)	32 (10%)	123 (40%)	106 (35%)	35 (11%)	9 (3%)	2 (1%)
Trait	Elasticity							Attitude					
Score	b	c	d	e	f	g	h	b	c	d	e		
Freq. (%)	7 (2%)	43 (14%)	102 (33%)	67 (22%)	62 (20%)	24 (8%)	2 (1%)	53 (17%)	165 (54%)	78 (25%)	10 (3%)		
Trait	Scope												
Score	b	c	d	e	f	g	h						
Freq. (%)	10 (3%)	56 (18%)	101 (33%)	73 (24%)	48 (16%)	18 (6%)	1 (0%)						

**Table 5:** Model 2; Regression coefficients in euros (€); error standard (SE) for factors (optimum and extreme trait scores) associated with potential event horse sales prices for 2022–2023.

Trait	Trait Score	Optimum/Non-Optimum Scores	Sales Price (€) b(SE)	*p*-Value
**Intercept**			−1,036,837 (821,259)	0.2078
**Head and Neck Connection**	1	opt: 3-non: 4–8	4813.99 (1068.34)	<0.0001
**Head and Neck Connection**	2		.	.
**Length of Neck**	1	opt: 3-non: 4–6	−164.12 (545.76)	0.7638
**Length of Neck**	2		.	.
**Position of Shoulder**	1	opt: 7-non: 3–6,8	1017.09 (760.34)	0.1821
**Position of Shoulder**	2		.	.
**Length of Croup**	1	opt: 3-non: 4–8	2814.57 (1205.44)	0.0202
**Length of Croup**	2		.	.
**Quality of Legs**	1	opt: 4-non: 3,5–8	4445.13 (921.19)	<0.0001
**Quality of Legs**	2		.	.
**Walk Length of Stride**	1	opt: 3-non: 1,2,4–7	2991.37 (897.09)	0.001
**Walk Length of Stride**	2		.	.
**Walk Correctness**	1	opt: 4-non: 2,3 5–8	−185.35 (596.01)	0.756
**Walk Correctness**	2		.	.
**Trot Elasticity**	1	opt: 2-non: 3–8	−7451.22 (3177.56)	0.0197
**Trot Elasticity**	2		.	.
**Trot Balance**	1	opt: 2-non: 3–8	3544.93 (973.72)	0.0003
**Trot Balance**	2		.	.
**Canter Length of Stride**	1	opt: 3-non: 2,4–9	−102.99 (534.86)	0.8474
**Canter Length of Stride**	2		.	.
**Canter Impulsion**	1	opt: 2-non: 3–8	−973.1 (977.17)	0.3202
**Canter Impulsion**	2		.	.
**Take off Direction**	1	opt: 2-non:3–8	1784.63 (711.06)	0.0126
**Take off Direction**	2		.	.
**Take off Quickness**	1	opt: 2-non: 3–8	−14.0214 (516.73)	0.9784
**Take off Quickness**	2		.	.
**Technique Back**	1	opt: 2-non: 3–8	506.6 (845.71)	0.5496
**Technique Back**	2		.	.
**Technique Haunches**	1	opt: 2-non: 3–8	−1204.34 (766.29)	0.1171
**Technique Haunches**	2		.	.
**Scope**	1	opt: 2-non: 3–8	8445.31 (1543.45)	<0.0001
**Scope**	2		.	.
**Care**	1	opt: 2-non: 3–8	−700.96 (554.63)	0.2073
**Care**	2		.	.

**Table 6:** Phenotypic traits Principal components, proportion of the explained variance (EV), and their cumulative sum (CEV) of principal components (PCs) 1 to 7 calculated 8 Phenotypic traits.

Eigenvectors, Eigenvalues, Proportion of the Explained Variance (EV), and Their Cumulative Sum (CEV) of Principal Components (PCs) 1 to 7 Calculated 8 Phenotypic Traits							
	PC1	PC2	PC3	PC4	PC5	PC6	PC7
Scope	0.38	−0.34	0.11	0.00	−0.34	−0.23	0.05
Elasticity	0.36	−0.48	−0.10	−0.07	−0.24	−0.34	−0.35
Canter Impulsion	0.37	0.14	0.04	−0.53	0.13	0.52	−0.51
Canter Balance	0.36	0.05	−0.46	−0.38	0.45	−0.28	0.47
Technique back	0.34	−0.40	−0.10	0.53	0.26	0.57	0.20
Canter Length of Stride	0.34	0.14	0.82	−0.07	0.10	−0.07	0.33
Trot Balance	0.34	0.46	−0.04	0.52	0.30	−0.34	−0.43
Trot Impulsion	0.34	0.49	−0.26	0.09	−0.66	0.20	0.25
Eigenvalue	4.895	0.868	0.529	0.493	0.364	0.357	0.286
EV%	61.19	10.85	6.61	6.16	4.55	4.46	3.58
CEV (%)	61.19	72.05	78.66	84.82	89.36	93.82	97.4

**Table 7:** Summary of traits strongly associated with each Principal Component (PC), the magnitude of the trait loading and their biological relevance.

Principal Component	Key Associated Traits	Trait Loading and Magnitude	Interpretation
PC1	Scope, Elasticity, Canter Impulsion.	(+0.38 to +0.36)	Athletic Scope, Jumping
PC2	Trot impulsion, Trot balance, Canter length of stride.	(+0.49 to +0.14)	Movement quality
PC3	Canter length of stride, Scope, Canter Impulsion.	(+0.82 to +0.04)	Jumping, Canter quality
PC4	Technique back, Trot balance, Trot impulsion.	(+0.53 to +0.09)	Trot and Jumping attributes
PC5	Canter Balance, Trot balance, Technique back.	(+0.45 to +0.26)	Jumping and balance
PC6	Technique back, Canter Impulsion, Trot impulsion.	(+0.57 to +0.20)	Jumping and Movement
PC7	Canter Balance, Canter length of stride, Trot impulsion.	(+0.47 to +0.25)	Locomotive Power

**Table 8:** Significant phenotypic traits influencing auction prices in young event horse auctions identified using two analytical models: full scores, binary optimum scores, and principal component analysis.

Linear Profile Trait	Model 1 All Scores Used	Model 2 Binary Opt./Non-Opt. Score	PCA
Head and Neck Connection	***	***	
Length of Neck			
Position of Shoulder			
Length of Croup		***	
Quality of Legs		***	
Walk Length of Stride	*	***	
Walk Correctness			
Trot Elasticity	*	***	
Trot Impulsion			PCA
Trot Balance	**	***	PCA
Canter Length of Stride			PCA
Canter Impulsion			PCA
Canter Balance			PCA
Take off Direction	*	***	
Take off Quickness			
Technique of Back			PCA
Technique of Haunches			
Elasticity			PCA
Scope	***	***	PCA
Care	***		

*p*-value, * *p* < 0.05, ** *p* < 0.01, *** *p* < 0.001.

## Data Availability

Restrictions apply to the availability of these data. Data were obtained from Irish Auction sales companies and may be available A F Corbally with the permission of each sales company.

## Abbreviations

The following abbreviations are used in this manuscript:

PCA	Principal Component Analysis
ISH	Irish Sport Horse
WBFSH	World Breeding Federation for Sport Horses
KWPN	Netherlands Royal Dutch Warmblood Studbook
VIF	Variance Inflation Factor
FEI	Federation Equestre Internationale

## Appendix A

**Table A1:** Selling Price variables by age, sex colour and height of horses for linearly scored phenotypic traits of young event horses sold at auction 2022–2023.

Variable	Level	Number of Horses	Mean Selling Price (€)	SD	95% Confidence Interval
Age	3	278	16,750	9205	15,663.2 to 17,836.8
	4	22	30,909	16,277	23,692.2 to 38,126
	5	7	26,000	8307	18,317.7 to 33,682.4
	Female	81	14,463	8143	12,662.5 to 16,263.5
Sex	Gelding	226	19,235	11,022	17,789.7 to 20,679.3
Colour	Bay	223	17,830	10,953	16,384.1 to 19,275.1
	Chestnut	39	16,769	8895	13,885.1 to 19,652.6
	Grey	37	19,473	10,200	16,072.1 to 22,873.8
	Other	8	21,000	7488	14,739.8 to 27,260.2
Height	164–172 cm	232	18,925	11,188	17,477.3 to 20,371.8
	173 cm	75	15,040	7557	13,301.2 to 16,778.8

**Table A2:** Model 1; Regression coefficients in euros (€); standard error (SE) on factors associated with potential event horse sales prices for 2022–2023.

Trait	Score	Sales Price (€) (SE)	*p* Value
Intercept		−2,255,719 (1,114,859)	0.0443
Head and Neck Connection	**3**	2053.88 (4250.84)	0.6295
	**4**	−2747.77 (4108.04)	0.5043
	**5**	−2840.67 (4100.12)	0.4892
	**6**	−3115.02 (4113.72)	0.4497
	**7**	−4981.12 (4157.22)	0.2322
	**8**	.	.
Length of Neck	**3**	−575.48 (2201.25)	0.794
	**4**	−613.67 (1537.46)	0.6902
	**5**	374.82 (1441.15)	0.795
	**6**	31.0103 (1484.69)	0.9834
	**7**	.	.
Position of Shoulder	**3**	2873.44 (4827.91)	0.5523
	**4**	2573.73 (4796.07)	0.5921
	**5**	1895.02 (4792.14)	0.6929
	**6**	1902.67 (4788.72)	0.6915
	**7**	2615.27 (4830.83)	0.5888
	**8**	.	.
Length of Croup	**3**	361.78 (5968.22)	0.9517
	**4**	−1124.01 (5865.48)	0.8482
	**5**	−1607.93 (5868.1)	0.7843
	**6**	−1823.46 (5843.44)	0.7553
	**7**	−79.0308 (5906.65)	0.9893
	**8**	.	.
Quality of Legs	**3**	−898.97 (2572.61)	0.7271
	**4**	2355.78 (2144.67)	0.2732
	**5**	−138.57 (1906.4)	0.9421
	**6**	53.4571 (2045.75)	0.9792
	**7**	.	.
Walk Length of Stride	**3**	2013.73 (2670.43)	0.4516
	**4**	−1414.94 (2485.08)	0.5697
	**5**	−951.49 (2491.05)	0.7029
	**6**	−53.6802 (2632.85)	0.9838
	**7**	.	.
Walk Correctness	**2**	4041.06 (4182.26)	0.335
	**3**	−847.25 (1748.52)	0.6285
	**4**	−680.79 (1609.69)	0.6728
	**5**	−1490.09 (1474.59)	0.3134
	**6**	−2394.3 (1612.76)	0.1391
	**7**	.	.
Trot Elasticity	**2**	−17,024 (5481.72)	0.0022
	**3**	−3823.83 (3233.01)	0.2382
	**4**	−3096.37 (3104.11)	0.3196
	**5**	−1527.28 (3121.01)	0.6251
	**6**	−1796.5 (3119.49)	0.5653
	**7**	−1875.42 (3278.5)	0.5679
	**8**	.	.
Trot Balance	**2**	7087.61 (9309.1)	0.4473
	**3**	4469.25 (1797.45)	0.0137
	**4**	1776.1 (1502.13)	0.2383
	**5**	−123.6 (1439.01)	0.9316
	**6**	33.1991 (1376.9)	0.9808
	**7**	.	.
Canter Length of Stride	**3**	−1430.05 (2602.84)	0.5833
	**4**	−1415.49 (2433.17)	0.5613
	**5**	−640.34 (2353.01)	0.7858
	**6**	−1071.38 (2331.51)	0.6463
	**7**	.	.
Canter Impulsion	**2**	8111.63 (6562.61)	0.2178
	**3**	−1982.3 (5028.62)	0.6938
	**4**	−749.04 (4998.59)	0.881
	**5**	−1503.19 (5001.78)	0.7641
	**6**	−927.2 (5047.57)	0.8544
	**7**	−1198.18 (5039.69)	0.8123
	**8**	.	.
Take off Direction	**2**	8293.7 (3282.61)	0.0122
	**3**	4268.11 (2447.41)	0.0826
	**4**	3202.7 (2394.86)	0.1825
	**5**	919.94 (2853.46)	0.7475
	**6**	1862.39 (2387.6)	0.4362
	**7**	1841.02 (2434.66)	0.4504
	**8**	.	.
Take off Quickness	**2**	4166.8 (2590.92)	0.1092
	**3**	−298.56 (1406.3)	0.8321
	**4**	1744.68 (1158.53)	0.1335
	**5**	810.99 (1284.71)	0.5285
	**6**	919.83 (1128.77)	0.416
	**7**	.	.
	**8**	0	.
Technique of Back	**2**	−11,719 (5003.92)	0.0201
	**3**	−2072.71 (2482.37)	0.4047
	**4**	−1758.49 (2386.31)	0.462
	**5**	−1428.6 (2380.97)	0.5491
	**6**	−819.07 (2295.14)	0.7215
	**7**	−1592.71 (2256.84)	0.4811
	**8**	.	.
Technique of Haunches	**2**	−4569.33 (2915.35)	0.1185
	**3**	−1002.65 (2523.68)	0.6915
	**4**	341.65 (2417.06)	0.8877
	**5**	762.78 (2446.75)	0.7555
	**6**	890.9 (2437.6)	0.7151
	**7**	−189.03 (2497.93)	0.9397
	**8**	.	.
Scope	**2**	5502.52 (5130.78)	0.2847
	**3**	−6566.21 (4836.8)	0.176
	**4**	−8142.19 (4762.75)	0.0888
	**5**	−7781.28 (4679.99)	0.0978
	**6**	−7304.58 (4619.4)	0.1153
	**7**	−6555.1 (4684.94)	0.1632
	**8**	.	.
Care	**2**	−1318.22 (3550.47)	0.7108
	**3**	−5014.16 (3376.44)	0.139
	**4**	−4914.09 (3344.53)	0.1432
	**5**	−2191.73 (3350.52)	0.5137
	**6**	−2223.27 (3494.82)	0.5253
	**7**	.	.
